# A Comprehensive Study on the Effect of TiN Top and Bottom Electrodes on Atomic Layer Deposited Ferroelectric Hf_0.5_Zr_0.5_O_2_ Thin Films

**DOI:** 10.3390/ma13132968

**Published:** 2020-07-02

**Authors:** Si Joon Kim, Jaidah Mohan, Harrison Sejoon Kim, Su Min Hwang, Namhun Kim, Yong Chan Jung, Akshay Sahota, Kihyun Kim, Hyun-Yong Yu, Pil-Ryung Cha, Chadwin D. Young, Rino Choi, Jinho Ahn, Jiyoung Kim

**Affiliations:** 1 Department of Electrical and Electronics Engineering, Kangwon National University, 1 Gangwondaehakgil, Chuncheon, Gangwon-do 24341, Korea; 2 Department of Materials Science and Engineering, The University of Texas at Dallas, 800 West Campbell Road, Richardson, TX 75080, USA; Jaidah.Mohan@utdallas.edu (J.M.); Harrison.Kim@utdallas.edu (H.S.K.); SuMin.Hwang@utdallas.edu (S.M.H.); namhun.kim@utdallas.edu (N.K.); yongchan.jung@utdallas.edu (Y.C.J.); kihyun.kim@utdallas.edu (K.K.); Chadwin.Young@utdallas.edu (C.D.Y.); 3 Department of Materials Science and Engineering, Inha University, 100 Inha-ro, Michuhol-gu, Incheon 22212, Korea; Rino.Choi@inha.ac.kr; 4 Department of Electrical and Computer Engineering, The University of Texas at Dallas, 800 West Campbell Road, Richardson, TX 75080, USA; Akshay.sahota@utdallas.edu; 5 School of Electrical Engineering, Korea University, 145 Anam-ro, Seongbuk-gu, Seoul 02841, Korea; yuhykr@korea.ac.kr; 6 School of Advanced Materials Engineering, Kookmin University, 77 Jeongneung-ro, Seongbuk-gu, Seoul 02707, Korea; cprdream@kookmin.ac.kr; 7 Division of Materials Science and Engineering, Hanyang University, 222 Wangshimni-ro, Seongdong-gu, Seoul 04763, Korea; jhahn@hanyang.ac.kr

**Keywords:** atomic layer deposition, ferroelectric film, Hf_0.5_Zr_0.5_O_2_, low thermal budget process, TiN electrode

## Abstract

The discovery of ferroelectricity in HfO_2_-based materials in 2011 provided new research directions and opportunities. In particular, for atomic layer deposited Hf_0.5_Zr_0.5_O_2_ (HZO) films, it is possible to obtain homogenous thin films with satisfactory ferroelectric properties at a low thermal budget process. Based on experiment demonstrations over the past 10 years, it is well known that HZO films show excellent ferroelectricity when sandwiched between TiN top and bottom electrodes. This work reports a comprehensive study on the effect of TiN top and bottom electrodes on the ferroelectric properties of HZO thin films (10 nm). Investigations showed that during HZO crystallization, the TiN bottom electrode promoted ferroelectric phase formation (by oxygen scavenging) and the TiN top electrode inhibited non-ferroelectric phase formation (by stress-induced crystallization). In addition, it was confirmed that the TiN top and bottom electrodes acted as a barrier layer to hydrogen diffusion into the HZO thin film during annealing in a hydrogen-containing atmosphere. These features make the TiN electrodes a useful strategy for improving and preserving the ferroelectric properties of HZO thin films for next-generation memory applications.

## 1. Introduction

The observation of unexpected ferroelectric properties in atomic layer deposited HfO_2_-based films in 2011 provided a pathway for new research directions and opportunities [1]. By selecting Zr among the various dopants reported, homogenous Hf_0.5_Zr_0.5_O_2_ (HZO) thin films having satisfactory ferroelectricity were realized using a low thermal budget process (400 °C) [2,3]. It has also been demonstrated that the thickness range of the feasible ferroelectric performance of HZO film can be reduced to 5 nm [4,5]. In this regard, numerous studies have been reported in various fields, such as ferroelectric random-access memory (FRAM), ferroelectric field-effect transistors, synaptic devices, and energy storage applications [6,7,8]. Based on theoretical reports and experimental demonstrations, the non-centrosymmetric orthorhombic phase (o-phase, space group: Pca2_1_) is now believed to be the reason for the origin of the ferroelectric behavior in these HZO films [6,7,8,9]. It should be noted that this o-phase is unusual because the stable crystallographic phase of HZO films is a non-polar monoclinic phase (m-phase) under typical semiconductor process conditions. To induce the ferroelectric o-phase formation with the suppression of the non-ferroelectric m-phase, the most important of the various factors is to provide a large tensile strain during crystallization of the HZO film [2,7,10]. It was also found that the applied stress depends on the type of electrode due to the difference in the thermal expansion coefficient [7,11]. In this regard, the influence of the top and bottom electrodes on HZO crystallization has been intensively studied, including the various electrode materials, the surface/interface effects, the capping layer effects, etc. [2,7,10,11,12,13]. Based on experiment demonstrations from the past 10 years, it has been established that HZO films exhibit excellent ferroelectricity in structures sandwiched with TiN top and bottom electrodes [7]. This implies that the stress from the TiN electrode during HZO crystallization and/or the interface between the TiN electrodes and the HZO film are beneficial in obtaining better ferroelectric performance. However, the reason for these aspects of the TiN electrode has not been clearly elucidated.

This study performed a comprehensive examination on the effect of TiN top and bottom electrodes on the ferroelectric properties of 10-nm-thick HZO films by adding or changing the order of the annealing process. In addition, the effects of the deposition temperature variation of the TiN top electrode and the substrate change for HZO deposition on the phase transformation of the HZO film were examined. Using the fabricated TiN/HZO/TiN capacitors, the influence of forming gas annealing (FGA), an inevitable process for obtaining an appropriate Si-based device performance, was also investigated.

## 2. Materials and Methods 

### 2.1. Sample Preparation

**Figure 1:**
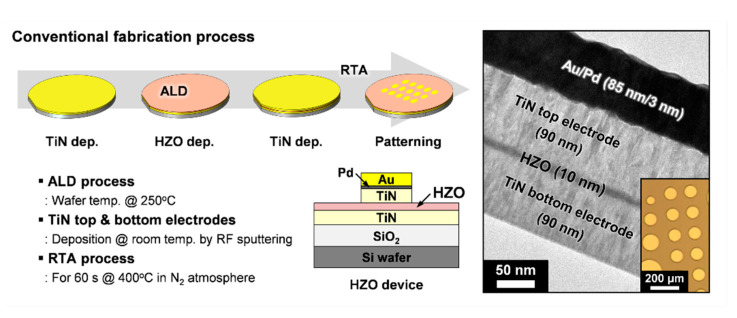
A schematic illustration of the conventional procedure used to fabricate MIM capacitors and cross-sectional transmission electron microscopy and optical microscopy images of the fabricated TiN/HZO/TiN capacitors.

The fabrication process as depicted in Figure 1 was identical to the methods that were described in our previous studies [2,3,4,5,14,15,16]. For the current work, metal-insulator-metal (MIM) capacitors were fabricated on 100 mm p-type Si wafers with a 300-nm-thick layer of thermally grown SiO_2_. The MIM structure consisted of 90-nm-thick TiN bottom and top electrodes deposited via radio frequency sputtering of a Ti target maintained at a power of 250 W. The Ti target was sputtered at room temperature in an environment of Ar and N_2_ gas with an Ar:N_2_ ratio of 20:1. An atomic layer deposition (ALD) method was adopted for depositing 10-nm-thick HZO films that contained Hf and Zr in a 1:1 ratio. The process was carried out at a wafer temperature of 250 °C using Savannah S100 ALD Cambridge Nanotech. In this process, Hf[N(CH_3_)_2_]_4_ (TDMA-Hf), Zr[N(CH_3_)_2_]_4_ (TDMA-Zr), and O_3_ were used as the Hf-precursor, Zr-precursor, and oxygen source, respectively. Since the ferroelectric properties of the HZO film depend on the composition and film thickness [6,7], the ALD technique was used in this study to precisely control these conditions. A high concentration of O_3_ (400 g/m^3^) was used in the process, which was obtained by an O_3_ generator (OP-250H, Toshiba Mitsubishi Electric Industrial Systems Corporation). The specific use of high concentration O_3_ with TDMA-Zr and TDMA-Hf at the given deposition temperature (250 °C) resulted in low carbon and hydrogen concentrations in the HZO film [14,15]. After the TiN top electrode was deposited, the annealing process was performed for 60 s at 400 °C in an N_2_ atmosphere using a rapid thermal annealing (RTA) system. Then, conventional photolithography and etching processes were performed using a gold hard mask (Au (85 nm)/Pd (3 nm)) deposited sequentially by an e-beam evaporator. The fabricated TiN/HZO/TiN capacitors with a diameter of 50–200 μm were confirmed by optical microscopy. In addition, the thickness of each layer, including the HZO film (10 nm) and the TiN top and bottom electrodes (90 nm), was confirmed by cross-sectional transmission electron microscopy (see Figure 1). These thickness values were chosen deliberately so as to obtain excellent ferroelectric properties as reported in previous studies [2,16]. Based on this conventional process, the effect of TiN top and bottom electrodes on ferroelectric HZO films was investigated by changing the order of the RTA process and/or performing an additional furnace annealing in hydrogen-containing ambience for 30 min at 400 °C (i.e., FGA).

### 2.2. Physical, Chemical, and Electrical Analysis

For estimating the stress caused by TiN top and bottom electrodes, the change in the radius of the curvature of the 100 mm wafers was measured (Toho FLX 2320-S, Toho Technology, Chicago, IL, USA) [17,18,19]. The crystal structures of the various HZO films were analyzed using a grazing-angle incidence X-ray diffraction (GIXRD, SmartLab, Rigaku, Tokyo, Japan) system in the 2θ range of 26°–40° with an incidence angle of 0.5°. Prior to GIXRD measurements, a wet-etch removal of the TiN top electrodes of all HZO samples was conducted using SC-1 (NH_4_OH + H_2_O_2_). To investigate the depth profile of the oxygen concentration, dynamic secondary ion mass spectrometry (D-SIMS, PHI ADEPT 1010, ULVAC-PHI, Chigasaki, Japan) with Cs as the primary ion source was employed.

For the electrical characterization, a semiconductor parameter analyzer (Keithley 4200-SCS, Keithley Instruments, Cleveland, OH, USA) at a 10 kHz frequency was used to measure the polarization-electric field (P-E) hysteresis curves. In addition, the pulse write/read test was performed using a pulse generator (Agilent 81110A, Agilent Technologies, Santa Clara, CA, USA) with an internal shunt resistor of 50 Ω resistance. A detailed description of the pulse write/read measurement method was provided in our previous studies [2,16]. To exhibit stable ferroelectric polarization, all HZO samples required a certain amount of wake-up cycles [4]. With the TiN top electrode given voltage bias and the bottom electrode kept grounded, 10^5^ wake-up cycles with a field of 2.5 MV/cm were performed.

## 3. Results and Discussion

In our previous studies, it was reported that by performing an annealing process for HZO crystallization after the deposition of the TiN top electrode, the TiN top electrode can act as a tensile stressor on the HZO film to stabilize the ferroelectric o-phase (known as the capping layer effect) [2]. However, there was a limitation in these results, as they do not completely exclude the stress effect from the TiN bottom electrode. To overcome these issues, in this study, the same RTA process was additionally performed after depositing the TiN bottom electrode (i.e., before the HZO deposition) to investigate only the stress caused by the TiN top electrode during HZO crystallization. Furthermore, the estimated stresses of the samples subjected to the RTA process for HZO crystallization before and after deposition of the TiN top electrode were also compared.

**Figure 2:**
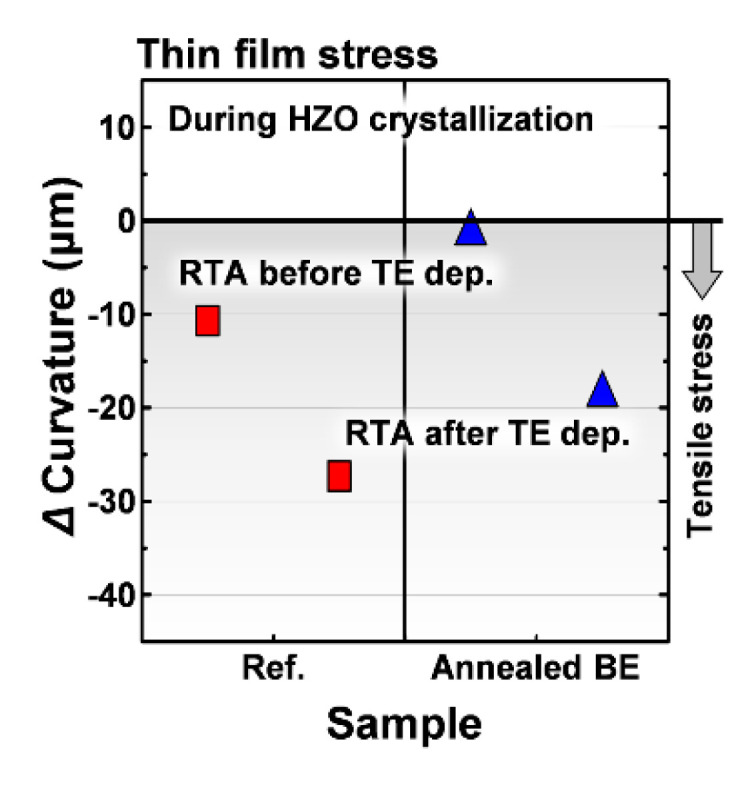
Variations in the radius of the curvature of the 100 mm wafers during HZO crystallization.

Figure 2 shows the curvature variations with (i.e., annealed BE) and without (i.e., Ref.) an RTA processed TiN bottom electrode, respectively. Based on the change in curvature of all samples, it was found that an in-plane tensile stress was generated in the HZO film regardless of the RTA process sequence. Meanwhile, when the RTA process was carried out after depositing the TiN top electrode, the estimated stress became larger. This trend was completely consistent with our previous studies [2,3,7].

**Figure 3:**
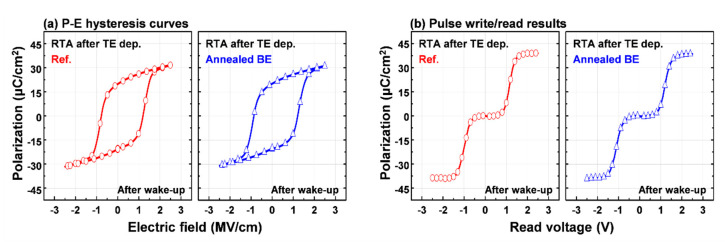
(**a**) The polarization-electric field hysteresis curves and (**b**) pulse write/read results of the 10-nm-thick HZO-based MIM capacitors annealed at 400 °C.

The P-E hysteresis and pulse write/read results of the 10-nm-thick HZO films that were deposited on the annealed (i.e., annealed BE) and non-annealed (i.e., Ref.) TiN bottom electrodes are shown in Figure 3, respectively. The RTA process for the crystallization of all HZO films was performed after TiN top electrode deposition (i.e., stress-induced crystallization [2]). After 10^5^ wake-up cycles, both HZO-based MIM capacitors exhibited similar double remnant polarization (2P_r_, about 40 μC/cm^2^), switching polarization (P_sw_, about 39 μC/cm^2^), and ferroelectric saturation voltage (V_sat_, about 1.5 V). These results indicate that the RTA process for the TiN bottom electrode made a difference in the applied stress during HZO crystallization (see Figure 2), but had no effect on the ferroelectric properties of the HZO-based MIM capacitors.

**Figure 4:**
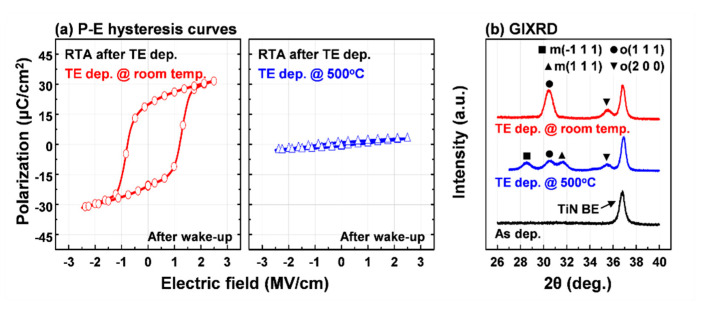
(**a**) The polarization-electric field hysteresis curves of 10-nm-thick HZO-based MIM capacitors with different deposition temperatures of TiN top electrodes; (**b**) GIXRD patterns of as-deposited HZO film and annealed HZO films after TiN top electrode deposition at room temperature and 500 °C, respectively.

In order to relieve stress from the top electrode, when the TiN top electrode was deposited at 500 °C, the ferroelectric properties were also investigated, as shown in Figure 4a. Although the RTA process for HZO crystallization was performed after depositing the TiN top electrode at 500 °C, the fabricated device exhibited linear dielectric properties. This is related to the formation of the m-phase in the HZO film confirmed by the GIXRD results (see Figure 4b). This m-phase formation may be due to the crystallization of the HZO film in the process of raising the temperature to deposit the TiN top electrode. Even in this case, this means that the TiN top electrode must be deposited before annealing in order to form the o-phase responsible for ferroelectric polarization. However, for the HZO film with TiN top electrode deposited at room temperature, m-phase diffraction peaks were not observed as in previous studies [2,14,16]. Consequently, the room-temperature deposited TiN top electrode can sufficiently act as the tensile stressor to the HZO film during the RTA process without the TiN bottom electrode.

**Figure 5:**
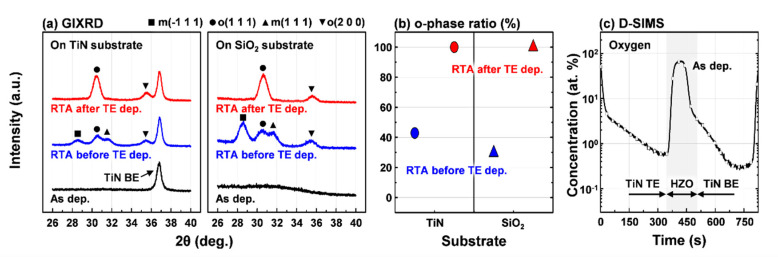
(**a**) The GIXRD patterns of 10-nm-thick HZO films on different substrates: as-deposited HZO films and HZO films annealed at 400 °C before and after TiN top electrode deposition; (**b**) the relative ratio of the area of o(1 1 1)/{m(-1 1 1)+o(1 1 1)+m(1 1 1)} of the HZO films calculated from the deconvoluted GIXRD patterns; (**c**) the D-SIMS depth profile of the as-deposited HZO film sandwiched between the TiN top and bottom electrodes.

Figure 5a shows the GIXRD patterns of 10-nm-thick HZO films on TiN and SiO_2_ substrates. Assumingly under identical stress from the TiN top electrode during the RTA process, the HZO films exhibited o-phase formation with inhibition of m-phase formation regardless of the type of substrate. This result suggests the feasibility of integrating a stress-induced crystallized HZO film into a metal-ferroelectric-insulator-semiconductor structure for steep slope devices. It also implies that the seed layer can be introduced as a bottom layer for depositing the HZO film [20]. Moreover, the HZO films that crystallized prior to the deposition of the TiN top electrode revealed the formation of both the o- and m-phases. The quantitative analyses of the relative portions of the o- and m-phases were attempted as shown in Figure 5b. The relative ratio of the integrated area of o(1 1 1)/{m(-1 1 1)+o(1 1 1)+m(1 1 1)} in the HZO films was calculated from the deconvoluted GIXRD patterns. For the HZO films annealed prior to TiN top electrode deposition, the relative ratio of the o(1 1 1)-phase of the HZO film on TiN substrate (~42.9%) was larger than that of the HZO film on SiO_2_ substrate (~29.7%). From these results, the role of the TiN bottom electrode for crystallization of the HZO film can be deduced. During the ALD process for the deposition of HZO film, the TiN bottom electrode was oxidized by a deposition temperature of 250 °C, which was confirmed through the depth profile of the oxygen concentration observed from the D-SIMS results in Figure 5c. This oxidation of the TiN bottom electrode can promote ferroelectric phase formation by increasing the oxygen vacancies at the interface between the TiN electrode and the HZO film [2,8,14,21]. This phenomenon, known as oxygen scavenging, can occur further during the RTA process. On the other hand, the oxygen vacancies remaining in the ferroelectric HZO film may degrade the reliability of the fabricated device [14,22,23]. Therefore, in order to obtain excellent reliability while maintaining a large ferroelectric polarization, an increase in oxygen vacancies at the interface is not necessarily desirable.

**Figure 6:**
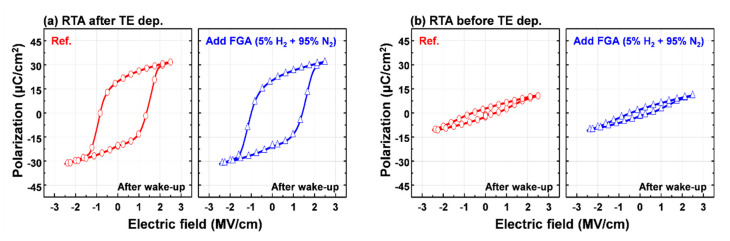
The polarization-electric field hysteresis curves of 10-nm-thick HZO-based MIM capacitors with and without additional FGA processes: HZO films annealed at 400 °C (**a**) after TiN top electrode deposition and (**b**) before TiN top electrode deposition.

Next, the influence of FGA on the ferroelectric properties of crystallized HZO films was examined. The P-E hysteresis curves of TiN/HZO/TiN capacitors before (i.e., Ref.) and after the FGA process are shown in Figure 6. FGA is an inevitable process for Si-based memory, but it is well known that FGA causes a severe degradation of the ferroelectric properties due to oxygen loss or hydrogen incorporation [24,25]. For this reason, FGA was one of the most critical processes that made the compatibility process of ferroelectric materials with Si CMOS difficult. However, TiN electrodes can act as a barrier layer to hydrogen diffusion into the ferroelectric HZO film [14,26]. As expected, there was no degradation in the ferroelectric properties observed in the HZO films using TiN electrodes due to FGA, which is consistent with previously reported studies [26,27]. In those studies, HZO films with Pt electrodes were reported to degrade ferroelectric properties during FGA due to hydrogen incorporation [26]. Meanwhile, the low 2P_r_ of the HZO film by non-ferroelectric m-phase formation also did not improve after FGA (see Figure 6b). These results make the ferroelectric HZO film sandwiched between the TiN top and bottom electrodes a suitable material for making a high density FRAM.

## 4. Conclusions

In conclusion, the effects of TiN top and bottom electrodes on the ferroelectric properties of atomic layer deposited HZO films were systematically investigated. The room-temperature deposited TiN top electrode was found to inhibit the non-ferroelectric m-phase formation by stress-induced crystallization during the RTA process. It was confirmed that this feature works regardless of the type of bottom layer for the deposition of the HZO film. However, a limitation occurred when the TiN top electrode was deposited at a high temperature. The TiN bottom electrode was found to increase oxygen vacancies at the interface by oxygen scavenging during HZO film deposition via the ALD process. These increased oxygen vacancies helped to form the ferroelectric o-phase during HZO crystallization. In addition, the HZO film sandwiched between the TiN top and bottom electrodes did not degrade the ferroelectric properties during annealing in a hydrogen-containing environment. Therefore, it can be concluded that the use of TiN top and bottom electrodes is a useful strategy for improving and preserving the ferroelectric properties of HZO films for next-generation memory applications.
